# Essential Oil and Non-Volatile Metabolites from *Kaunia longipetiolata* (Sch.Bip. ex Rusby) R. M. King and H. Rob., an Andean Plant Native to Southern Ecuador

**DOI:** 10.3390/plants11212972

**Published:** 2022-11-03

**Authors:** Omar Malagón, Cinthia Bravo, Giovanni Vidari, Nixon Cumbicus, Gianluca Gilardoni

**Affiliations:** 1 Departamento de Química, Universidad Técnica Particular de Loja (UTPL), Calle Marcelino Champagnat s/n, Loja 110107, Ecuador; 2 Department of Medical Analysis, Faculty of Applied Science, Tishk International University, Erbil 44001, Kurdistan Region, Iraq; 3 Dipartimento di Chimica, Università di Pavia, 27100 Pavia, Italy; 4 Departamento de Ciencias Biológicas, Universidad Técnica Particular de Loja (UTPL), Calle Marcelino Champagnat s/n, Loja 110107, Ecuador

**Keywords:** *Ageratina longipetiolata*, *Eupatorium longipetiolatum*, *Eupatorium uber*, *Kaunia arbuscularis*, *Kaunia uber*, Asteraceae, essential oil, enantioselective analysis, guaianolides, dehydroleucodine

## Abstract

*Kaunia longipetiolata* (Sch.Bip. ex Rusby) R. M. King and H. Rob. (Asteraceae) is a plant native to southern Ecuador. The dry leaves afforded, by steam distillation, an essential oil that was qualitatively and quantitatively analyzed by GC-MS and GC-FID, respectively, on two orthogonal columns of different polarity. Sesquiterpenes predominated in the volatile fraction, among which α-zingiberene (19.7–19.1%), *ar*-curcumene (17.3–18.1%), caryophyllene oxide (5.1–5.3%), (*Z*)-β-caryophyllene (3.0–3.1%), (2*Z*,6*Z*)-farnesal (2.6–3.6%), and spathulenol (2.0–2.1%) were the major components. In addition to the identified compounds, two main unidentified constituents (possibly oxygenated sesquiterpenes) with probable molecular masses of 292 and 230, respectively, were detected. They constituted about 5% and 8% (*w/w*), respectively, of the whole essential oil. The oil chemical composition was complemented with the enantioselective analysis of ten chiral components. Four scalemic mixtures and six enantiomerically pure terpenes were identified. An enantiomeric excess (*ee*) was determined for (1*R*,5*R*)-(+)-β-pinene (65.0%), (*R*)-(−)-α-phellandrene (94.6%), (*S)*-(+)-linalool (15.0%), and (*R*)-(−)-terpinen-4-ol (33.8%). On the other hand, (1*R*,5*R*)-(+)-α-pinene, (1*R*,5*R*)-(+)-sabinene, (*S*)-(−)-limonene, (*S*)-(+)-β-phellandrene, (1*R*,2*S*,6*S*,7*S*,8*S*)-(−)-α-copaene, and (*R*)-(+)-germacrene D were enantiomerically pure. Finally, the non-volatile fraction obtained by extraction of the leaves with MeOH was investigated. Eight known compounds were isolated by liquid column chromatographic separations. Their structures were determined by NMR spectroscopy as dehydroleucodine, kauniolide, (3*S*,3a*R*,4a*R*,6a*S*,9a*S*,9b*R*)-3-hydroxy-1,4a-dimethyl-7-methylene-5,6,6a,7,9a,9b-hexahydro-3H-oxireno[2′,3′:8,8a]azuleno[4,5-b]furan-8(4aH)-one, novanin, bisabola-1,10-diene-3,4-*trans*-diol, (*R*)-2-(2-(acetoxymethyl)oxiran-2-yl)-5-methylphenyl isobutyrate, eupalitin-3-*O*-glucoside, and 3,5-di-*O*-caffeoylquinic acid. Literature data about the identified metabolites indicate that *K. longipetiolata* is a rich source of biologically active natural products.

## 1. Introduction

Medicinal plants can historically be considered the first source of effective drugs, whose knowledge is an important heritage of many traditional cultures worldwide. The biological properties of vegetal drugs are mainly attributed to the presence of specialized metabolites, whose structural and biological relationships with human endogenous biomolecules can explain their pharmacological activities. However, pharmacology is not the only interesting application of natural products, since several specialized metabolites are components of volatile fractions, which are typically responsible for the scent of aromatic plants. Upon submission to steam distillation, aromatic species afford an organic phase called essential oil (EO) that usually spontaneously separates from water. Most of these volatile oils are important commercial products, widely used by pharmaceutical, cosmetic, food, and household detergency industries.

Currently, most of the European and North American flora has been phytochemically investigated and the search for new natural products is focused on the plants of tropical countries. In these regions, the existence of a rich biodiversity together with a historically scarce scientific investigation make the discovery of new natural products quite promising [1,2]. In particular, the so-called “megadiverse” countries are invaluable sources of phytochemically unprecedented botanical species. In fact, according to the UN Environment Programme, these countries are characterized by possessing two-thirds of all non-fish vertebrate and three-fourths of all higher plant species of the world [3]. Due to the rich and unique biodiversity, Ecuador is included among the megadiverse countries. For this reason, our group has been investigating for many years the phytochemistry of the Ecuadorian flora, which is largely unexplored. Our aims are to discover new biologically active natural products and to contribute to the advancements of scientific knowledge concerning the neotropical flora [4,5,6,7,8,9,10]. More recently, we focused especially on the study of EOs, since they can be interesting sources of sesquiterpenoids, bioactive and flavoring products, as well as novel mixtures of enantiomers with different enantiomeric excesses [11,12,13,14,15,16,17].

**Figure 1:**
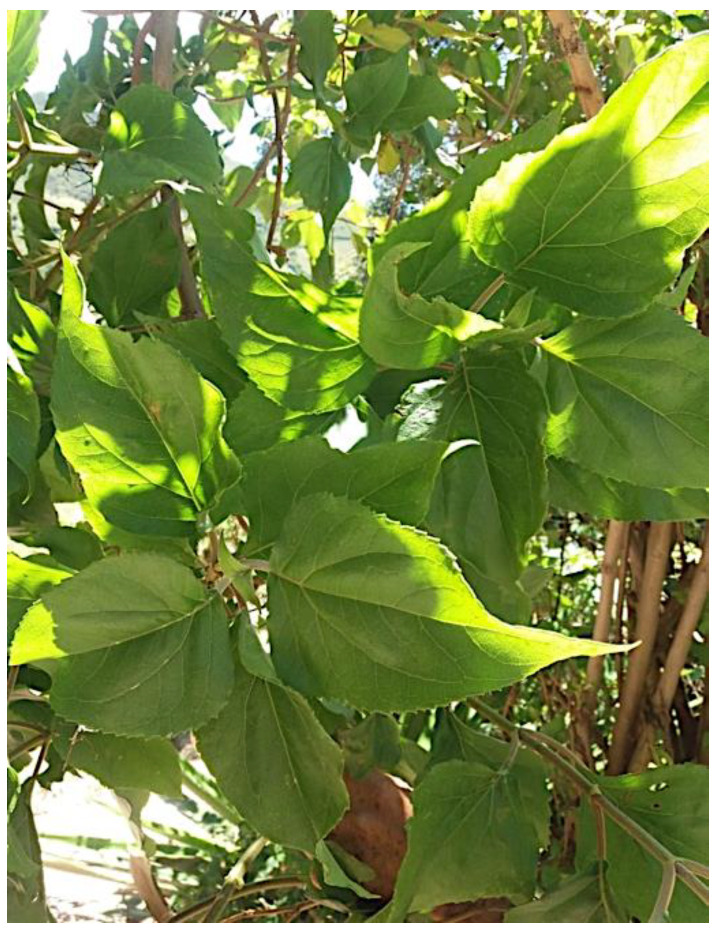
*Kaunia longipetiolata* (Sch.Bip. ex Rusby) R. M. King and H. Rob. Photo by Cinthia Bravo.

In this context, *Kaunia longipetiolata* (Sch.Bip. ex Rusby) R. M. King and H. Rob. (Figure 1) was selected as a promising plant for the study of its EO. In fact, this species belongs to the family Asteraceae, which is well known for the abundance of EO-bearing taxa. Despite the wide distribution of the plant, no study has been reported about an EO from *K. longipetiolata*. Furthermore, the phytochemistry of the entire genus *Kaunia* is very little described in literature. The structures of non-volatile constituents from the aerial parts of *K. arbuscularis* (B. L. Robins) R. M. King and H. Rob., *K. ignorata* (Hieron.) R. M. King and H. Rob., *K. saltensis* (Hieron.) R. M. King and H. Rob., and *K. lasiophthalma* (Griseb.) R. M. King and H. Rob., including several sesquiterpene guaianolides, have been reported in a few studies [18,19,20]. However, no biological activity or traditional medicinal use is described in literature for *K. longipetiolata*.

In the present work, the chemical and enantiomeric composition of the EO from leaves of *K. longipetiolata* are described for the first time. The major components of the EO were α−zingiberene, *ar*-curcumene, caryophyllene oxide, (*Z*)-β-caryophyllene, (2*Z*,6*Z*)-farnesal, and spathulenol. This study was completed with the investigation of a non-volatile fraction, extracted from another lot of leaves of *K. longipetiolata*. Three known guaianolides (**13**–**15**), including high amounts of dehydroleucodine, together with five other known compounds (**16**–**20**), were isolated. 

From the botanical point of view, *Kaunia* R. M. King and H. Rob. (Asteraceae, Eupatorieae) is a small genus of about ten species, comprising shrubs or small trees that play an important role in the Andean ecosystems, from Ecuador to northwestern Argentina and, exceptionally, Brazil and Paraguay [21,22]. They are mostly distributed in different environments of subtropical forests, especially in shrubby layers or secondary forests [22,23]. The native distribution of *K. longipetiolata* ranges from Bolivia to southern Ecuador, where it occurs in different bioclimatic zones such as sub-humid, pluviseasonal, and mesotropical to dry and xeric, at an altitude ranging from 1000 to 4000 m above sea level [22,23]. The plant is a very common tree in the province of Loja (Ecuador), where it is used for tracing borders between rural properties [21]. It is interesting to note that *K. longipetiolata* (Rusby) R. M. King and H. Rob. and *K. arbuscularis* (B. L. Rob.) R. M. King and H. Rob. are considered two separate accepted species in the list of vascular plants appearing in the World Flora Online, while they have been considered heterotypic synonyms in a recent revision of the genus *Kaunia* [22,24]. *K. longipetiolata* is also known by the synonyms of *Ageratina longipetiolata* (Rusby) R. M. King and H. Rob., *Eupatorium longepetiolatum* Sch. Bip., *Eupatorium longipetiolatum* Rusby, *Eupatorium longipetiolatum var. longipetiolatum, Eupatorium uber* B. L. Rob., and *Kaunia uber* (B. L. Rob.) R. M. King and H. Rob. [24].

## 2. Results

### 2.1. EO from Leaves of K. longipetiolata

#### 2.1.1. Chemical Analysis 

The EO was distilled from the leaves of *K. longipetiolata*, with an average yield of 0.13 ± 0.05% (*w/w*), calculated over four analytical repetitions. The volatile fraction was composed mainly of sesquiterpenes, corresponding to 81.9% and 84.8% of the whole EO, analyzed by GC-FID on a non-polar and polar column, respectively. To the entire sesquiterpene fraction, hydrocarbons contributed for 44.3% and 44.8% (*w/w*), respectively, whereas oxygenated derivatives were 37.6% and 40.0% (*w/w*). A total of 72 GC peaks were detected, of which 69 corresponded to individual oil components identified on at least one column. Instead, two likely oxygenated sesquiterpenoids, together accounting for 14.2% and 12.1% (*w/w*), were unidentified, whereas a third GC peak corresponded to a mixture of three unseparated unidentified compounds. The two main unknown compounds, with probable molecular weights of 292 and 230 (Table 1), respectively, were separated as a mixture from the whole EO (obtained by a preparative distillation). However, they were inseparable from each other by both normal-phase and reversed-phase liquid chromatography. 

**Figure 2:**
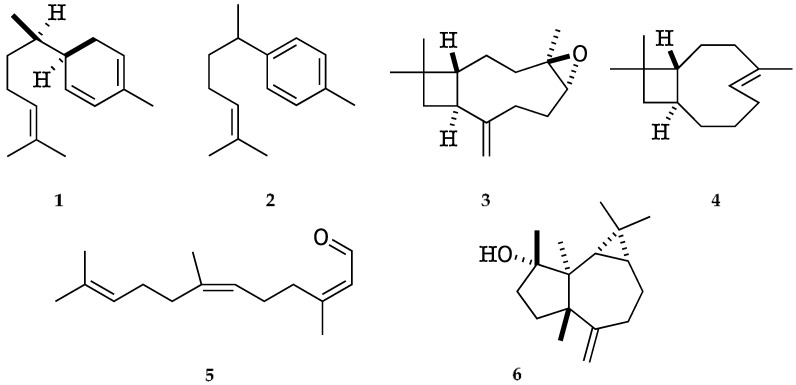
Major identified compounds in the EO of *K. longipetiolata*.

**Figure 3:**
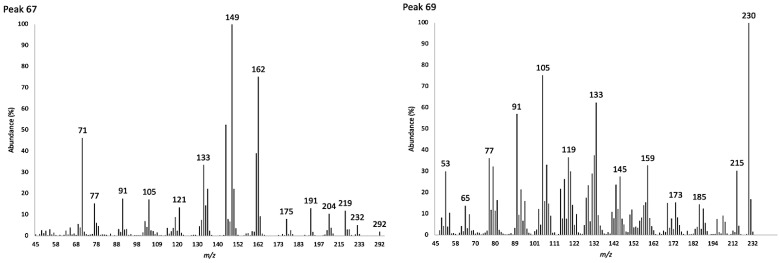
Electronic impact mass spectra of the unidentified compounds corresponding to peaks 67 (**left**) and 69 (**right**).

**Figure 4:**
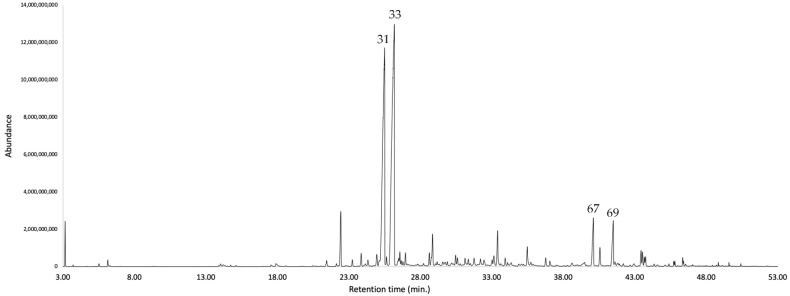
GC-MS chromatogram of *K. longipetiolata* EO on a 5%-phenyl-methylpolysiloxane stationary phase column. The peaks are numbered according to the order of oil components listed in Table 1.

**Figure 5:**
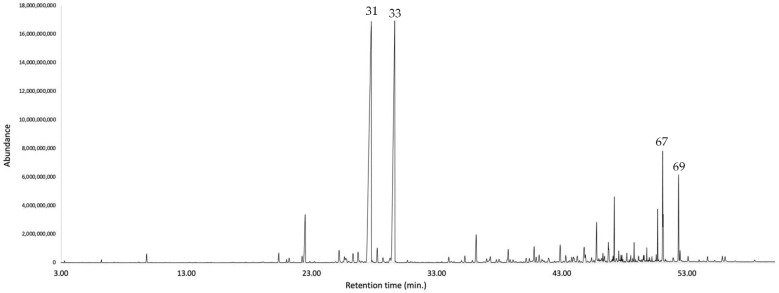
GC-MS chromatogram of *K. longipetiolata* EO on a polyethylene glycol stationary phase column. The peaks are numbered according to the order of oil components listed in Table 1.

The major identified compounds were α-zingiberene (**1**) (19.7–19.1% of the EO, *w/w*), *ar*-curcumene (**2**) (17.3–18.1%), caryophyllene oxide (**3**) (5.3–5.1%), (*Z*)-β-caryophyllene (**4**) (3.0–3.1%), (2*Z*,6*Z*)-farnesal (**5**) (2.6–3.6%), and spathulenol (**6**) (2.0–2.1%). Their chemical structures with the corresponding numbers in bold are depicted in Figure 2. All the quantified constituents accounted for 87.5% and 92.5% of the whole EO, calculated as the sum of the integrated peaks with respect to total area of the gas chromatogram. A relative response factor was calculated for each oil component (see Section 4.4.1) and the resulting corrected chromatographic areas were interpolated using a calibration curve. The qualitative and quantitative composition of the EO from *K. longipetiolata* is reported in Table 1, whereas the mass spectra of the two main unidentified compounds and the GC-MS chromatograms on the two columns are shown in Figure 3, Figure 4 and Figure 5.

**Table 1:** Chemical analysis of *Kaunia longipetiolata* essential oil on 5%-phenyl-methylpolysiloxane and polyethylene glycol GC columns. The progressive numbers are referred to peaks in Figure 4 and Figure 5.

N.	Compounds	5%-Phenyl-Methylpolysiloxane				Polyethylene Glycol				
		LRI ^a^	LRI ^b^	%	σ	LRI ^c^	LRI	Reference	%	σ
1	α-pinene	925	932	0.3	0.01	1018	1025	[25]	1.1	0.99
2	sabinene	963	969	Trace	-	1119	1122	[25]	Trace	-
3	β-pinene	968	974	Trace	-	1108	1110	[25]	0.1	0.01
4	dehydro-1.8-cineole	986	988	0.2	0.10	1188	1187	[26]	0.1	0.01
5	δ-2-carene	995	1001	0.2	0.07	1128	1133	[25]	0.2	0.01
6	α-phellandrene	1003	1002	0.2	0.01	1162	1167	[25]	0.2	0.14
7	*p*-cymene	1020	1020	0.3	0.15	1270	1270	[25]	0.3	0.20
8	limonene	1024	1024	Trace	-	1198	1198	[25]	Trace	-
9	β-phellandrene	1043	1025	Trace	-	1207	1209	[25]	Trace	-
10	(*E*)-β-ocimene	1052	1044	Trace	-	1253	1250	[25]	Trace	-
11	terpinolene	1080	1086	Trace	-	1281	1282	[25]	0.1	0.01
12	α-gurjunene	-	-	-	-	1519	1529	[25]	Trace	-
13	linalool	1104	1095	0.1	0.01	1553	1543	[25]	0.2	0.06
14	terpinen-4-ol	1177	1174	0.2	0.06	1601	1601	[25]	Trace	-
15	coahuilensol, methyl ether	1218	1219	0.4	0.18	1674	-	-	0.1	0.05
16	thymol, methyl ether	1228	1232	Trace	-	1596	1587	[25]	0.2	0.01
17	carvacrol, methyl ether	1236	1241	Trace	-	1607	1599	[25]	0.1	0.01
18	thymol	1292	1289	0.4	0.13	2179	2164	[25]	1.6	0.48
19	carvacrol	1300	1298	0.7	0.30	2195	2210	[25]	1.2	0.46
20	α-copaene	1363	1374	Trace	-	1482	1491	[25]	Trace	-
21	2-*epi*-α-funebrene	1380	1380	0.2	0.08	1533	-	-	0.2	0.08
22	sesquithujene	1396	1405	Trace	-	1563	1560	[27]	0.2	0.06
23	(*Z*)-β-caryophyllene (4)	1405	1408	3.0	0.55	1585	1588	[25]	3.1	0.26
24	α-*trans*-bergamotene	1425	1432	0.2	0.10	1579	1575	[25]	0.2	0.08
25	alloaromadendrene	1433	1432 ^d^	Trace	-	1651	1649	[25]	0.1	0.01
26	α-humulene	1440	1452	0.9	0.26	1658	1667	[25]	0.9	0.28
27	(*E*)-β-farnesene	1452	1454	0.2	0.12	1669	1664	[25]	0.2	0.10
28	germacrene D	1466	1481	0.9	0.57	1698	1708	[25]	1.4	0.36
29	γ-curcumene	1471	1481	0.3	0.13	1687	1692	[25]	0.3	0.17
30	α-selinene	-	-	-	-	1710	1725	[25]	0.1	0.01
31	*ar*-curcumene (2)	1477	1479	17.3	5.31	1772	1773	[25]	18.1	4.95
32	10-*epi*-β-acoradiene	1481	1474	0.4	0.08	1722	-	-	0.2	0.05
33	α-zingiberene (1)	1491	1493	19.7	11.36	1718	1721	[25]	19.1	7.68
34	sesquicineole	1505	1515	0.5	0.19	1736	1733	[28]	0.7	0.29
35	δ-cadinene	1507	1522	0.4	0.11	1750	1756	[25]	0.4	0.18
36	β-curcumene	1510	1514	0.2	0.05	1724	1737	[25]	Trace	-
37	β-sesquiphellandrene	1516	1521	0.3	0.17	1764	1771	[25]	0.3	0.15
38	γ-cuprenene	1538	1532	0.3	0.14	-	-	-	-	-
39	*cis*-sesquisabinene hydrate	1549	1542	0.2	0.08	2112	2110	[29]	1.5	0.38
40	(*E*)-nerolidol	1560	1561	1.0	0.21	2048	2036	[25]	1.2	0.40
41	Spathulenol (6)	1564	1577	2.0	0.99	2117	2127	[25]	2.1	1.03
42	(*E*)-β-ionone	-	-	-	-	1934	1936	[25]	0.3	0.15
43	caryophyllene oxide (3)	1566	1558	5.3	2.55	1969	1966	[30]	5.1	2.34
44	ar-turmerol	1574	1582	0.5	0.15	2212	2214	[31]	0.6	0.24
45	salvial-4(14)-en-1-one	1578	1594	0.7	0.00	1995	1995	[32]	0.5	0.14
46	humulene epoxide II	1593	1608	1.0	0.51	2025	2026	[29]	1.3	0.65
47	α-acorenol	1616	1632	0.3	0.05	2169	2163	[25]	0.1	0.06
48	isospathulenol	-	-	-	-	2224	2230	[25]	0.3	0.06
49	β-atlantol	1619	1608	0.3	0.13	2229	-	-	1.0	0.32
50	gossonorol	1632	1636	0.8	0.22	2308	2312	[33]	1.2	0.38
51	*epi*-α-muurolol	1643	1640	1.0	0.37	2143	2153	[34]	0.2	0.08
52	14-hydroxy-9-*epi*-(*E*)-caryophyllene	1659	1668	0.4	0.07	1962	-	-	0.6	0.44
53	*epi*-β-bisabolol	1662	1670	0.6	0.25	2149	2150	[35]	0.7	0.19
54	khusinol	1671	1679	0.4	0.16	2290	-	-	0.8	0.44
55	eudesma-4(15),7-dien-1β-ol	1676	1687	0.8	0.31	2357	2371	[25]	1.0	1.00
56	*epi*-α-bisabolol	1677	1683	0.2	0.10	2217	2214	[25]	0.4	0.08
57	α-bisabolol	1680	1685	1.1	0.50	2220	2213	[25]	0.2	0.05
58	(*2Z,6Z*)-farnesal (5)	1686	1684	2.6	0.44	2270	-	-	3.6	2.03
59	γ- costol	-	-	-	-	2516	-	-	0.3	0.13
60	curcuphenol	1716	1717	0.7	0.29	2617	-	-	0.8	0.49
61	cryptomerione	1730	1724	0.3	0.15	2480	-	-	0.6	0.23
62	(*6R,7R*)-bisabolone	1737	1740	0.4	0.22	2297	-	-	0.2	0.09
63	xanthorrhizol	1748	1751	1.2	0.36	2678	2674	[32]	1.5	0.30
64	β-costol	1756	1765	0.5	0.22	2578	-	-	0.5	0.25
65	α-costol	1761	1773	0.4	0.17	2574	-	-	0.4	0.18
66	(*Z*)-nuciferol acetate	1821	1830	0.2	0.07	2337	-	-	1.1	0.45
67	Unidentified (MW 232)	1882	-	5.2	1.30	2714	-	-	4.8	0.99
68	Unidentified (mixture)	1897	-	0.6	0.38	2794	-	-	0.5	0.18
69	Unidentified (MW 230)	1925	-	9.0	4.51	2831	-	-	7.3	1.84
70	palmitic acid	1972	1971 ^e^	2.3	2.15	2844	-	-	-	-
71	phytol	2111	2103 ^f^	0.3	0.10	2597	2603	[36]	0.8	0.46
72	sempervirol	2285	2282	Trace	-	-	-	-	Trace	-
	*Monoterpene hydrocarbons*			*1.0*					*2.0*	
	*Oxygenated monoterpenes*			*1.9*					*3.5*	
	*Sesquiterpene hydrocarbons*			*44.3*					*44.8*	
	*Oxygenated sesquiterpenes*			*37.6*					*40.0*	
	*Others*			*2.7*					*2.2*	
	*Total identified*			*87.5*					*92.5*	

^a^ Calculated linear retention indices on DB5-MS capillary column; ^b^ linear retention indices according to literature [37]. ^c^ Calculated linear retention indices on HP-INNOWax capillary column; ^d^ [38], ^e^ [39], ^f^ [40]. Traces ≤ 0.1%. MW = molecular weight.

#### 2.1.2. Enantioselective GC Analysis 

**Figure 6:**
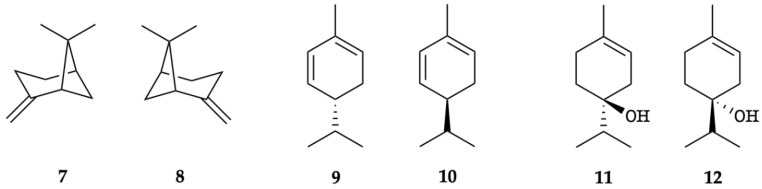
Enantiomeric pairs detected by GC enantioselective analysis: (1*R*,5*R*)-(+)-β-pinene (**7**), (1*S*,5*S*)-(–)-β-pinene (**8**), (*R*)-(−)-α-phellandrene (**9**), (*S*)-(+)-α-phellandrene (**10**), (*S*)-(+)-terpinen-4-ol (**11**), and (*R*)-(−)-terpinen-4-ol (**12**).

The enantioselective GC analysis of *K. longipetiolata* EO indicated ten chiral components, of which six were enantiomerically pure, whereas four occurred as scalemic mixtures (**7**/**8**, **9**/**10**, and **11**/**12**, see Figure 6). The enantiomeric distribution and the enantiomeric excess (*ee*) of these chiral terpenoids are shown in Table 2.

### 2.2. Non-Volatile Metabolites from Leaves of K. longipetiolata 

**Figure 7:**
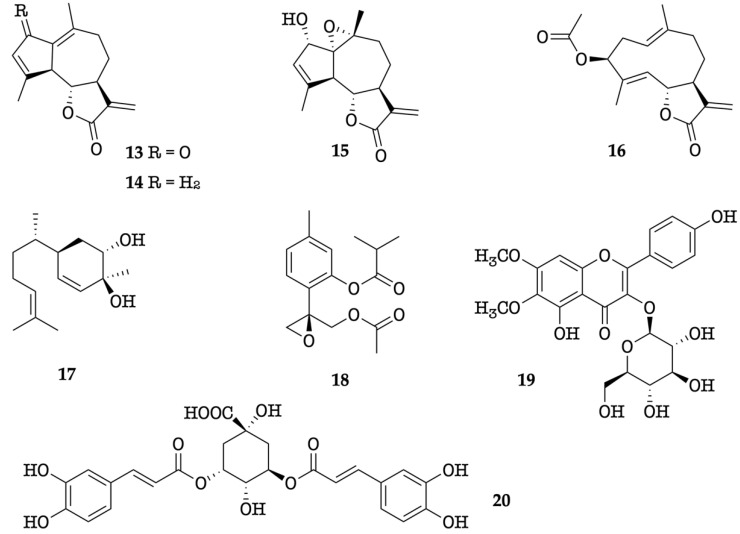
Non-volatile secondary metabolites isolated from the leaves of *K. longipetiolata* and identified by NMR spectroscopy. The numbers are referred to in Section 1, Section 2 and Section 3.

**Figure 8:**
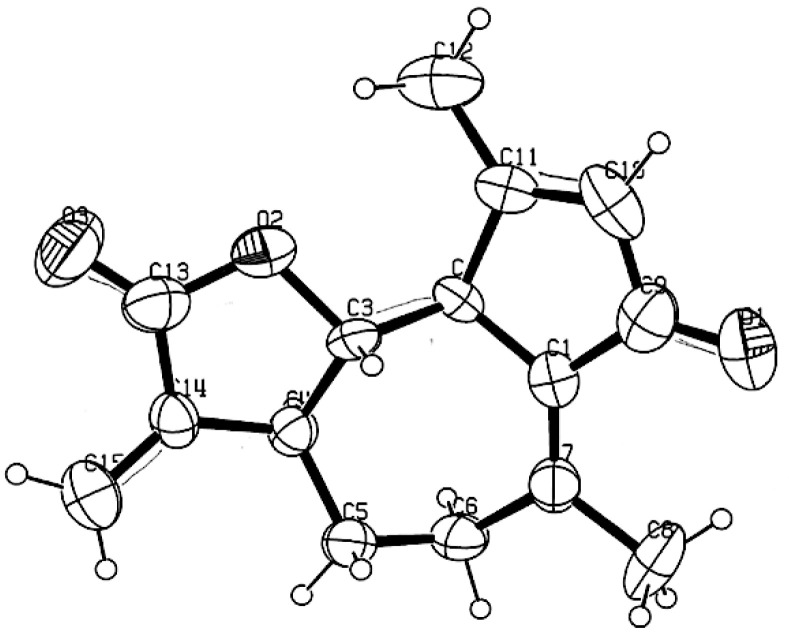
Computer-generated Oak Ridge Thermal Ellipsoid Plot (ORTEP) of dehydroleucodine (**13**) from X-ray diffraction analysis.

The methanol extract of *K. longipetiolata* dry leaves, after solvent partition and preparative liquid chromatography, afforded eight specialized compounds, whose numbered chemical structures are depicted in Figure 7. Depending on their structure and biogenesis, three of them belonged to the family of guaianolide sesquiterpenoids (**13–15**), whereas the others included a germacranolide sesquiterpene (**16**), a bisabolene derivative (**17**), a monoterpenoid thymol derivative (**18**), a glucosyl flavonol (**19**), and a shikimic acid derivative dicaffeoyl ester (**20**). The isolation of these compounds is already described in the literature: dehydroleucodine (**13**) from *K. arbuscularis* [18], *K. ignorata* [18], and *K. lasiophthalma* [20]; kauniolide (**14**) from *K. arbuscularis* [18]; the lactone **15** from *K. lasiophthalma* [20]; novanin (**16**) from *K. arbuscularis* [19], a few *Artemisia* species [41,42,43], and from *Argyranthemum adauctum* [44]; bisabola-1,10-dien-3,4-*trans*-diol (=3,4-*trans*-dihydroxybisabola-1,10-diene) (**17**) from *Alpinia densibracteata* [45]; the diester **18** from *K. ignorata* [18]; and the rare flavonoid eupalitin-3-*O*-glucoside (**19**) from *Sesuvium portulacastrum* [46]. Finally, 3,5-di-*O*-caffeoylquinic acid (**20**) is a common natural product, occurring in several widespread plants, such as *Pyrus communis* and *Ipomoea batatas* [47,48]. The structures of compounds **13–20** were determined from NMR data which were compared with those reported in the literature. Furthermore, the X-ray diffraction analysis of easily crystallizable dehydroleucodine (**13**) afforded the ORTEP structure shown in Figure 8.

## 3. Discussion

The EO from leaves of *K. longipetiolata* contained an abundant sesquiterpene fraction, where two compounds, α-zingiberene (**1**) and *ar*-curcumene (**2**) (Figure 2), accounted for about 37% (*w/w*) of the whole oil mass. Due to their biogenesis, the compounds **1** and **2** are usually found together in the EOs of several plants, where they are typically major components. This is the case of the *Zingiber* species [49,50,51], whose EOs are endowed with antibacterial, antifungal, antioxidant, and anti-inflammatory activities [52,53]. Interestingly, the amounts of compounds **1** and **2** in the EO distilled from the rhizomes of *Z. officinale* were comparable to those determined in the EO of *K. longipetiolata* [49]. Isolated α-zingiberene exhibited moderate cytotoxic activity against HeLa, U-87, Siha, and HL60 cell lines, and apoptotic effects the in the cervix cancer cell SiHa [54,55]. Moreover, α-zingiberene (**1**) showed high anti-rhinoviral activity in vitro [56] and therapeutic potential in the treatment of pathologies in which processes such as inflammation and angiogenesis are exacerbated, and even for the treatment of chronic wounds [57]. On the other hand, *ar*-curcumene (**2**) was identified as one of the main compounds responsible for the antitumor effects of *Curcuma zanthorrhiza* [58], and as an antiulcer [59] and anti-inflammatory agent of ginger [60]. In fact, it reduced the lipopolysaccharide (LPS)-induced secretion of the proinflammatory chemokine interleukin 8 (IL-8) in human bronchial epithelial cells [61]. Compound **2** also showed high larvicidal and oviposition deterrence activity against malaria, dengue, Zika, filariasis, and St. Louis encephalitis mosquito vectors [60]. 

Regarding the non-volatile metabolites, sesquiterpenoid lactones **13**–**16** are of high biological interest. In fact, sesquiterpenoids incorporating a cyclic enone and/or α,β-unsaturated lactone moieties can react readily as electrophiles with nucleophilic sites of biomacromolecules [62,63]. As a result, they exhibit a wide range of biological activities such as anti-inflammatory, antiparasitic, antibacterial, and antiproliferative effects [64]. Guaianolides are mainly occurring in the Asteraceae and Apiaceae families. Dehydroleucodine (**13**) (synonyms: 11,13-dehydroleucodin, dehydroleucodin, leucodin, lidbeckialactone, mesatlantin E), isolated from *Kaunia* [18,19,20], *Artemisia* [42,65], and *Stevia* [66] species (Asteraceae), showed gastroduodenal mucosal protection in response to necrosis-inducing agents, inhibition of chemical-induced mast cell degranulation, neuropeptide-induced mast cell activation, and cytotoxicity in mouse melanoma cells and a mouse melanoma model, human astrocytoma, and leukemia cells [65]. Recently, it was demonstrated that dehydroleucodine (**13**) possessed DNA methyl transferase (DNMT) inhibitory activity, reduced global and gene-specific DNA methylation, and increased expression of tumor suppressor genes *MHL1* and *PTEN*, which can contribute to the induction of apoptosis and cell death. It was concluded that lactone **13** holds the exciting potential as a novel therapeutic agent in ovarian cancer [65]. The cytotoxicity of dehydroleucodine (**13**) in vitro and in vivo has also been the subject of intense studies in our laboratories [67,68,69,70,71,72], leading the authors to patent its application for treatment of cancer [73]. It is important to emphasize that, in the present study, dehydroleucodine was obtained in high yields (>3.8% *w/w*) from *K. longipetiolata* dry leaves, and it was easily purified by spontaneous crystallization from a chromatographic fraction (see Section 4.4.3). Moreover, *K. longipetiolata* is widely distributed in southern Ecuador, and it is not protected and is easily cultivable. For all these features, the plant can be considered a new excellent source of the promising drug dehydroleucodine (13).

As concerns the compounds **14**–**20**, they have been less studied, and no important biological activity has yet been reported for them. However, due to their rareness, some of them are interesting chemotaxonomically. This is, for instance, the case of compound **15**, isolated so far only from *K. lasiophthalma* [20] and the thymol derivative **18**, previously found in *K. longipetiolata* [18] and *Eupatorium glechonophyllum* [74].

**Scheme 1:**
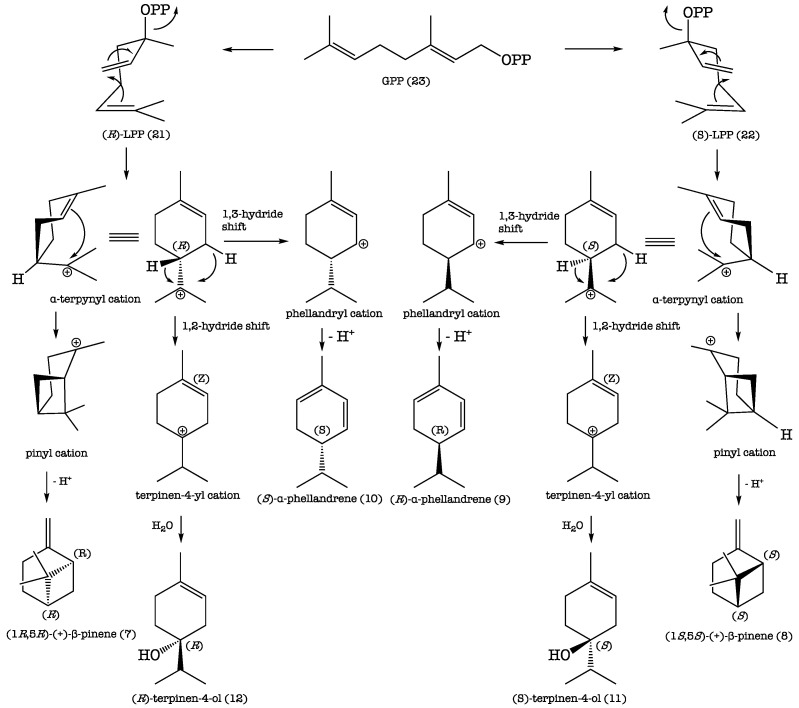
Proposed biosynthetic pathways of the couples of enantiomers **7**/**8**, **9**/**10**, and **11**/**12**.

The results of the GC enantioselective analysis deserve a separate comment. The enantioselective analysis of the EO was carried out using an enantioselective capillary column, based on modified β-cyclodextrines. Cyclodextrines are chiral cyclic polysaccharides, whose internal cavity can host small chiral molecules adding dissymmetric information. For this reason, the host–guest interaction presents a diastereomeric character, allowing two enantiomers to be separated. In the present study, the finding that a few terpenoid components of the EO occurred as scalemic mixtures (Table 2) indicated that the biochemical machinery of *K. longipetiolata* contained two separate enzyme systems, each capable of elaborating a single enantiomer [75]. Thus, the formation of the separate enantiomers of linalyl pyrophosphate (LPP), (*R*)-**21** and (*S*)-**22**, from geranyl pyrophosphate (GPP) (**23**), followed by analogous carbocation reactions, would explain the production of the couples of monoterpene enantiomers **7**/**8**, **9**/**10**, and **11**/**12**, as shown in Scheme 1.

## 4. Materials and Methods

### 4.1. Plant Material

The leaves of *K. longipetiolata* were collected on two different dates from plants growing in the same location: in March 2003 for the study of the non-volatile fraction, and in June 2018 for the investigation of the EO. The time gap between the two botanical collections is because the investigation of non-volatile metabolites was carried out in Italy in 2005, while the analysis of the EO was conducted in Ecuador in 2018. For the sake of completeness, the results of the two studies are reported together in this paper. The collection site corresponded to an area of about 0.5 km^2^, around a central point of coordinates 3°51′44.1′′ S and 79°11′46.8′′ W, in the province of Loja (southern Ecuador). Vouchers are deposited at the herbarium of the Universidad Técnica Particular de Loja (UTPL), with accession code HUTPL 1345. This investigation was carried out under the permission of the Ministry of Environment, Water and Ecological Transition of Ecuador, with MAATE registry number MAE-DNB-CM-2016-0048. The botanical species was identified by one of the authors (N.C.). The leaves were dried at 35° for 48 h and stored in a dark and fresh place until use.

### 4.2. Distillation of the EO

The essential oil used for the qualitative and quantitative analyses was obtained by analytical steam distillation of dry leaves (100 g) in a Marcusson-type apparatus for 4 h. The process was repeated four times. To collect the oil, a solution (2.0 mL) containing *n*-nonane as the internal standard (6.8 mg) in cyclohexane (10 mL) was introduced in the collection tube, above the aqueous phase. The resulting cyclohexane solution was directly injected in the GC instrument. Additionally, for the attempted separation of the unidentified components, the EO was steam-distilled for 4 h in a stainless-steel Clevenger-type apparatus, obtaining 1.4 g of a pure essential oil from 1.1 Kg of dry plant material. The yield and chemical composition of the pure EO were the same as those obtained by the analytical process.

### 4.3. Extract Preparation

Dry leaves (1.2 Kg) of *K. longipetiolata* were submitted to exhaustive solvent extraction with methanol (MeOH, 7.5 L) by repeated maceration at room temperature. Subsequently, the solvent was removed by under vacuum distillation. The total extract (12.2 g) was partitioned between hexane (Hex) and acetonitrile (MeCN), producing two immiscible phases and a residue (R, 6.5 g). The evaporated polar phase (4.5 g) was submitted to solid-phase extraction (SPE) to remove residual chlorophylls and other non-polar compounds. In this process, the acetonitrile fraction was eluted with 85% aqueous methanol through a 45 g reversed-phase column, affording 3.2 g of a chlorophyll-free polar extract.

### 4.4. Determination of Chemical Compositions

The chemical and enantioselective qualitative analyses of the EO were carried out on an Agilent Technologies GC-MS instrument, consisting of a 6890N gas chromatograph coupled to a mass spectrometry detector (MSD), model 5973 INERT (Santa Clara, CA, USA). The GC was additionally equipped with a common flame ionization detector (FID) that was used for the EO quantitative analysis. The MSD was set in SCAN mode (scan range 40–350 *m/z*), with the electron ionization (EI) source set at 70 eV. The GC qualitative and quantitative analyses were run on both a non-polar and a polar capillary column. The non-polar column (DB-5ms from Agilent Technologies, 30 m long, 0.25 mm internal diameter, and 0.25 μm film thickness) was based on a 5%-phenyl-methylpolysiloxane stationary phase. The polar column (HP-INNOWax, from Agilent Technologies, 30 m × 0.25 mm × 0.25 μm) was based on a polyethylene glycol stationary phase. The GC enantioselective analysis was conducted on a capillary column, based on 2,3-diethyl-6-*tert*-butyldimethylsilyl-β-cyclodextrin as the chiral selector. The enantioselective column (25 m × 0.250 mm internal diameter × 0.25 μm phase thickness) was purchased from Mega, Milan, Italy. The carrier gas for all the analyses was helium (GC purity grade from Indura, Guayaquil, Ecuador), set at the constant flow rate of 1 mL/min.

The preparative chromatographic separations of the non-volatile fraction were carried out on open columns at atmospheric pressure (CC). The columns were manually packed with silica gel (Merck Kieselgel 60, 40–63 μm, Kenilworth, NJ, USA) or C_18_ reversed phase (Merck LiChroprep RP-18, 25–40 μm), both purchased from Sigma-Aldrich (St. Louis, MO, USA). All thin-layer chromatographic (TLC) analyses were conducted over silica gel 60 (0.25 mm; GF_254_, Merck) or RP-18 (F254s, Merck) plates (Sigma-Aldrich). TLC plates were initially visualized under UV light (254 and 366 nm); subsequently, they were sprayed with a 0.5% solution of vanillin in H_2_SO_4_/ethanol 4:1, and finally heated at 200 °C. For chlorophyll removal, solid-phase extraction (SPE) was carried out in a glass column, manually packed with the same reversed phase cited above. All NMR experiments were carried out with Bruker 300 MHz and Bruker 500 MHz spectrometers (Bruker, Billerica, MA, USA). All deuterated solvents were purchased from Sigma-Aldrich. Single crystal X-ray diffraction experiments were carried out on an Enraf-Nonius four-circle diffractometer, model CAD-4 (Enraf-Nonius B.V., Rotterdam, The Netherlands), applying a graphite monochromated Mo-Kα radiation. The generated ORTEP structure of dehydroleucodine (**13**) (Figure 5) shows 50% probability displacement ellipsoids. Previously distilled technical grade solvents were used for preparative CC, whereas HPLC-grade solvents (Sigma-Aldrich) were used for all other applications. The mixture of *n*-alkanes C_9_–C_25_ and the internal standard (*n*-nonane) for GC analyses were analytical grade (purity > 99%), purchased from Sigma-Aldrich. The calibration standard was isopropyl caproate, synthesized by one of the authors (G.G.) and purified to 98.8% purity (GC-FID).

Semi-preparative HPLC was carried out on a Jasco PU-2080 PLUS dual pump HPLC instrument, equipped with a UV detector (JASCO Corporation, Tokyo, Japan). The eluent flow was set at 10 mL/min, whereas the UV detector was operated at 215 nm. The sample was injected through a 1.5 mL loop.

#### 4.4.1. GC-MS and GC-FID Analyses

For qualitative analyses of EOs, GC-MS is currently the technique of choice, since very similar volatile compounds can usually be separated with good resolution on a capillary gas chromatographic (GC) column. On the other hand, mass spectrometry (MS) is a detection method that provides information on the structure of an analyte and allows its identification through the analysis of the mass spectrum. The GC-FID technique is used for quantitative analysis since the flame ionization detector (FID) produces relative response factors (RRFs) dependent on the molecular formula and the presence of aromatic rings in the analyte structure. 

The qualitative and quantitative analyses of the EO were conducted on both the non-polar and polar columns, using the same temperature program: initially, 60 °C for 5 min, followed by a thermal gradient of 3 °C/min until 180 °C, followed by a second ramp of 10 °C/min until 250 °C, which was maintained for 5 min. The injections were performed in split mode (40:1), with an injection volume of 1 µL. For each chromatographic peak, the linear retention index (LRI) was calculated according to Van Den Dool and Kratz, referred to a mixture of a homologous series of *n*-alkanes (C_9_–C_25_) [76]. The constituents of the volatile fraction were identified by comparing their MS spectra and LRI values with the literature. For quantification, a relative response factor (RRF) was at first calculated for each oil component, referred to isopropyl caproate as the calibration standard. The RRFs were mathematically obtained with the assumption that in GC-FID, under the above-mentioned conditions, they only depend on combustion enthalpy [77,78]. Subsequently, the content of each component in the EO was calculated from the corrected area of the corresponding peak, which was interpolated using a calibration curve, traced for each column [16]. Both curves showed a correlation coefficient of 0.995. Finally, the analytical yield of the EO was calculated from the total amount of the volatile fraction, which corresponded to the sum of the areas of all peaks in the gas chromatogram, corrected by a mean RRF value.

#### 4.4.2. Enantioselective GC Analysis

The enantioselective analysis was carried out by GC-MS, since β-cyclodextrin-based capillary columns allow to separate volatile enantiomers with high resolution, whereas mass spectrometry permits to confirm the presence of enantiomeric pairs, due to the identity of their mass spectra. The enantioselective analysis was performed using the same method and instrument configuration as the qualitative analyses, except for the oven temperature program that was as follows: initially, 60 °C for 5 min, followed by a gradient of 2 °C/min up to 220 °C, which were maintained for 2 min. In addition, in this case, the LRIs were calculated according to Van Den Dool and Kratz [76]. The elution order of the enantiomers was determined by injecting enantiomerically pure standards.

#### 4.4.3. Metabolite Purification and NMR Analysis

The chlorophyll-free polar extract (2.3 g) was separated through a column of silica gel (200 g), eluted with a gradient of Hex and ethyl acetate (EtOAc), from 3% to 50% EtOAc, for a total of 1.6 L. After TLC analysis, the collected fractions (8 mL each) with similar composition were brought together, obtaining 24 main fractions, from OM-1 to OM-24. From fraction OM-12, dehydroleucodine (**13**, 337 mg) spontaneously crystallized as the most abundant metabolite. Subsequently, novanin (**16**, 20 mg) was separated from fraction OM-8 by reversed-phase chromatography (isocratic 85% aqueous MeOH).

The fractions from OM-1 to OM-3 were brought together (33.1 mg) and separated by a column of silica gel (3 g). Elution with Hex-EtOAc, from 0% until 50% EtOAc, afforded pure kauniolide (**14**, 2 mg).

A mixture of two or more components of very similar polarity are usually inseparable by standard CC. In this case, high-performance liquid chromatography (HPLC) can be used due to its higher chromatographic resolution. For this reason, the fractions from OM-13 to OM-15 were brought together (110.2 mg) and separated on an HPLC-UV instrument, using a hand-packed semi-preparative reversed-phase column (45 g). The eluent was a mixture of MeCN-H_2_O (30:70), held for 3 min, followed by a 17 min increasing gradient until 100% MeCN, held for 20 min. Compounds **15** (3.0 mg) and **17** (5.6 mg) were isolated in a pure form.

Another HPLC-UV separation of OM-4 (56.9 mg), carried out with MeCN-H_2_O mixtures, from 20% MeCN (3 min) to 100% MeCN in 20 min, afforded compound **18** (23 mg).

Finally, the residue R described in Section 4.3. was chromatographed on a column of silica gel (250 g). Elution with a polarity gradient, from 100% EtOAc to EtOAc-MeCO_2_H-HCO_2_H-H_2_O (76:8:8:8), afforded pure 3,5-di-*O*-caffeoylquinic acid (**20**, 198.9 mg) and semi-purified eupalitin-3-*O*-glucoside (**19**). Reversed-phase chromatography with a gradient of aqueous MeOH finally afforded pure compound **19** (7.6 mg). Subsequently, as is customary in phytochemical studies, all the purified compounds were submitted to nuclear magnetic resonance (NMR) and MS spectrometry analysis, and the chemical structures were elucidated by spectra interpretation. All the isolated compounds **13**–**20** resulted to have already been described in the literature, and their ^1^H and ^13^C NMR spectra were identical with published data [18,19,20,45,46,47,48]. As regards dehydroleucodine (**13**), X-ray diffraction was also chosen as the structure elucidation method, due to the spontaneous tendency of this sesquiterpene lactone to produce high-quality crystals. 

## 5. Conclusions

The leaves of *K. longipetiolata*, a plant native to Ecuador, afforded by steam distillation an essential oil not yet investigated, with a yield of 0.13% (*w/w*) in dry plant material. Due to the chemical composition, especially for the presence of high amounts of α-zingiberene (**1**) and *ar*-curcumene (**2**), the oil has a therapeutic potential in the treatment of pathologies, in which processes such as inflammation and angiogenesis are exacerbated. Enantioselective analysis of the EO indicated that the biosynthetic pathways, leading to a few terpenoid components, involved two separate enzyme systems, each capable of elaborating a single enantiomer. Moreover, the non-volatile fraction of *K. longipetiolata* proved to be a rich source of the antitumor guaianolide dehydroleucodine (**13**), which could be easily purified with a yield > 3.8% (*w/w*) on the total methanol extract.

## Figures and Tables

**Table 2:** Enantioselective analysis of *K. longipetiolata* EO on a 2,3-diethyl-6-*tert*-butyldimethylsilyl-β-cyclodextrin-based GC column.

LRIs	Enantiomers	Enantiomeric Distribution (%)	*ee* (%)
932	(1*R*,5*R*)-(+)-α-pinene	100	100
960	(1*R*,5*R*)-(+)-β-pinene (**7**)	82.5	65.0
969	(1*S*,5*S*)-(−)-β-pinene (**8**)	17.5	
989	(1*R*,5*R*)-(+)-sabinene	100	100
1029	(*R*)-(−)-α-phellandrene (**9**)	97.3	94.6
1036	(*S*)-(+)-α-phellandrene (**10**)	2.7	
1064	(*S*)-(−)-limonene	100	100
1070	(*S*)-(+)-β-phellandrene	100	100
1199	(*R)-*(−)-linalool	42.5	15.0
1209	(*S)*-(+)-linalool	57.5	
1270	(*S*)-(+)-terpinen-4-ol (**11**)	33.1	33.8
1272	(*R*)-(−)-terpinen-4-ol (**12**)	66.9	
1332	(1*R*,2*S*,6*S*,7*S*,8*S*)-(−)-α-copaene	100	100
1473	(*R*)-(+)-germacrene D	100	100

## Data Availability

Raw data are available from the authors (O.M. and C.B.)
